# Retraction: Newcastle disease virus fusion protein is the major contributor to protective immunity of genotype-matched vaccine

**DOI:** 10.1371/journal.pone.0244074

**Published:** 2020-12-14

**Authors:** The PLOS ONE Editors

After this article [1] was published, concerns were raised about results reported in Figs 2A and 3A.

Specifically:

- The Mock-infected panel of Fig 2A in [1] appears similar to the Mock-infected panel of Fig 3A in [2]. The authors confirmed that they used the same image to represent the mock-control in both publications. They stated that the same DF1 cells were used as a control for both of the experiments, and that per their observations there is little difference in batch-to-batch results for mock-infected DF1 cells.
- There to be a vertical discontinuity separating the lanes 1–4 from lane 5 in the top panel of Fig 3A. The authors attributed this to a smiling effect due to the voltage setting used during electrophoresis and commented that the data were obtained from a single SDS-PAGE gel.

The authors noted that the original data underlying the results reported in this article are no longer available. There are seven instances where the Results section refers to data not shown; the data underlying those statements are likewise not available.

This article was included in an investigation by the Department of Health and Human Services (HHS), which concluded there was evidence of research misconduct in the case of the first issue described above [3].

In light of the above concerns, the unavailability of the original data, and the outcome of the HHS investigation, the *PLOS ONE* Editors retract this article.

SKS agreed with retraction. S-HK did not agree with retraction and stands by the article’s findings. PLC agreed with the retraction, stands by the article's findings, and apologizes for the issues with the published article. The other authors either could not be reached or did not respond directly to comment on the retraction decision.

## References

[1] Kim S-H, Wanasen N, Paldurai A, Xiao S, Collins PL, Samal SK (2013) Newcastle Disease Virus Fusion Protein Is the Major Contributor to Protective Immunity of Genotype-Matched Vaccine. PLoS ONE 8(8): e74022 10.1371/journal.pone.0074022 24015313 PMC3755997

[2] Kim S-H, Xiao S, Shive H, Collins PL, Samal SK (2013) Mutations in the Fusion Protein Cleavage Site of Avian Paramyxovirus Serotype 4 Confer Increased Replication and Syncytium Formation In Vitro but Not Increased Replication and Pathogenicity in Chickens and Ducks. PLoS ONE 8(1): e50598 10.1371/journal.pone.0050598 23341874 PMC3544850

[3] (2020) FR Doc. 2020–10253. Federal Register 85(93): 28643–28645. PMC7220845 32435075

